# Intrinsic Deregulation of Vascular Smooth Muscle and Myofibroblast Differentiation in Mesenchymal Stromal Cells from Patients with Systemic Sclerosis

**DOI:** 10.1371/journal.pone.0153101

**Published:** 2016-04-07

**Authors:** Björn Hegner, Theres Schaub, Rusan Catar, Angelika Kusch, Philine Wagner, Kirill Essin, Claudia Lange, Gabriela Riemekasten, Duska Dragun

**Affiliations:** 1 Clinic for Nephrology and Intensive Care Medicine, Charité University Hospital, Berlin, Germany; 2 Berlin-Brandenburg School for Regenerative Therapies (BSRT), Berlin, Germany; 3 Center for Cardiovascular Research (CCR), Charitè University Hospital, Berlin, Germany; 4 Institute for Chemistry and Biochemistry, Freie Universität Berlin, Berlin, Germany; 5 Experimental and Clinical Research Center, Charité Medical Faculty and the Max-Delbrück Center for Molecular Medicine, Berlin, Germany; 6 Clinic for Stem Cell Transplantation, Department of Cell and Gene Therapy, University Medical Center Hamburg-Eppendorf, Hamburg, Germany; 7 Clinic for Rheumatology and Clinical Immunology, Charité University Hospital, Berlin, Germany; National Cancer Institute, UNITED STATES

## Abstract

**Introduction:**

Obliterative vasculopathy and fibrosis are hallmarks of systemic sclerosis (SSc), a severe systemic autoimmune disease. Bone marrow-derived mesenchymal stromal cells (MSCs) from SSc patients may harbor disease-specific abnormalities. We hypothesized disturbed vascular smooth muscle cell (VSMC) differentiation with increased propensity towards myofibroblast differentiation in response to SSc-microenvironment defining growth factors and determined responsible mechanisms.

**Methods:**

We studied responses of multipotent MSCs from SSc-patients (SSc-MSCs) and healthy controls (H-MSCs) to long-term exposure to CTGF, b-FGF, PDGF-BB or TGF-β1. Differentiation towards VSMC and myofibroblast lineages was analyzed on phenotypic, biochemical, and functional levels. Intracellular signaling studies included analysis of TGF-β receptor regulation, SMAD, AKT, ERK1/2 and autocrine loops.

**Results:**

VSMC differentiation towards both, contractile and synthetic VSMC phenotypes in response to CTGF and b-FGF was disturbed in SSc-MSCs. H-MSCs and SSc-MSCs responded equally to PDGF-BB with prototypic fibroblastic differentiation. TGF-β1 initiated myofibroblast differentiation in both cell types, yet with striking phenotypic and functional differences: In relation to H-MSC-derived myofibroblasts induced by TGF-β1, those obtained from SSc-MSCs expressed more contractile proteins, migrated towards TGF-β1, had low proliferative capacity, and secreted higher amounts of collagen paralleled by reduced MMP expression. Higher levels of TGF-β receptor 1 and enhanced canonical and noncanonical TGF-β signaling in SSc-MSCs accompanied aberrant differentiation response of SSc-MSCs in comparison to H-MSCs.

**Conclusions:**

Deregulated VSMC differentiation with a shift towards myofibroblast differentiation expands the concept of disturbed endogenous regenerative capacity of MSCs from SSc patients. Disease related intrinsic hyperresponsiveness to TGF-β1 with increased collagen production may represent one responsible mechanism. Better understanding of repair barriers and harnessing beneficial differentiation processes in MSCs could widen options of autologous MSC application in SSc patients.

## Introduction

Systemic sclerosis (SSc) is a complex progressive multisystem disorder featuring vasculopathy, autoimmunity and extensive fibrosis of skin and organs [1]. SSc vasculopathy includes both, microvascular and macrovascular changes. While capillary rarefaction is a morphologic denominator of microvascular changes [2], occlusive macrovasculopathy of arterioles and arteries features excessive neointima formation in parallel to medial and adventitial fibrosis [3]. Current concepts suggest that failure of vascular regeneration with an inappropriate local tissue healing response may result in uncontrolled deposition of extracellular matrix which is central to the pathogenesis of SSc [4].

Malfunctioning precursor and mature cells types contribute to this combination of defective maintenance of vascular integrity and adverse pro-fibrotic tissue remodeling in response to cytokine and growth factor (GF) microenvironmental stimuli. Recent data implicate defects in endothelial cell progenitor (EPC) numbers and functions [5] together with SSc-related hyporesponsiveness to pro-angiogenic stimuli. Constitutively activated myofibroblasts derived from lesional skin or fibrotic lungs from affected patients with excessive collagen production are another paradigmatic example [1, 6]. Mesenchymal stromal cells (MSC) from SSc patients have preserved expansion capacities, mesenchymal differentiation abilities, and immunomodulatory properties [7], yet show defective differentiation towards endothelial lineage [8]. Although MSCs are a potential myofibroblast source in fibroproliferative diseases [1], SSc specific changes at the interface between myofibroblast and phenotypically overlapping vascular smooth muscle cell (VSMC) differentiation have not been studied. VSMCs and myofibroblasts share many phenotypic features [9]. Similarly to VSMCs, MSCs bear a high potential for neointimal growth due to their phenotypic plasticity [10, 11].

We hypothesized that multipotent bone marrow derived MSCs from SSc patients (SSc-MSCs) harbor intrinsic differentiation abnormalities comprising the VSMC-myofibroblast axis in response to disease associated microenviroment favoring a phenotypic switch towards myofibroblasts. We compared features of phenotypic VSMC-myofibroblast conversion of MSCs from healthy controls (H-MSCs) and SSc-MSCs in response to key mediators including connective tissue growth factor (CTGF), basic fibroblast growth factor (b-FGF), platelet derived growth factor-BB (PDGF-BB) and transforming growth factor-β1 (TGF-β1). To better understand mechanisms responsible for phenoconversion of MSCs into SSc lesional cell types we addressed differences in receptor expression, signaling pathways, and autocrine regulation.

## Methods

### Patients and controls

We assayed MSCs from six representative patients with SSc and from six age- and sex-matched healthy controls. Patients were between 38 and 74 years (median 50) of age; four were women (67%). All patients had digital ulcers and suffered from pulmonary and skin fibrosis. Three had limited cutaneous SSc, three had diffuse cutaneous SSc (dSSc) with a disease duration of 11–120 month (median 92). All individuals with dSSc were positive for Scl-70 antibodies, the others were either positive for anti-centromer or U1-RNP antibodies or had no detectable auto-antibodies. Four patients received intermittent corticosteroid therapy. Controls were healthy subjects without any sign of autoimmune or fibrotic diseases who donated bone marrow for allogeneic transplantation. The study protocol was approved by the local institutional review board (Ethikkomission der Charité –Universitätsmedizin Berlin). All subjects were included in the study after providing written informed consent.

### Isolation and culture of MSC

Up to 10 ml aspirated iliac crest bone marrow were diluted 1:2 with PBS (PAA) and layered onto Percoll (density 1.124 g/ml; Biochrom, Berlin, Germany) diluted to a density of 1.068 g/ml. After centrifugation at 800g for 20 min at room temperature, mononuclear cells (MNC) were collected from the interphase, washed twice with PBS, and plated at a density of 4x10^4^/cm^2^ in α-MEM (#E15-862, PAA) supplemented with 100 U/mL penicillin (PAA), 100 μg/mL streptomycin (PAA), 2 IU/ml heparin (Ratiopharm), and 5% freshly thawed platelet lysate [12]. Cells were incubated at 37°C and 5% CO_2_. Non-adherent cells were washed off with PBS after 2–3 days. Medium was changed twice a week.

When cultures reached about 90% confluence, cells were detached with 0.05% Trypsin/0.02% EDTA (PAA), counted, and re-plated at 500 cells/cm^2^ in 175 cm^2^ flasks (Saarstedt). Under these culture conditions, MSC maintain multilineage differentiation capacity, express characteristic surface marker proteins (CD59, CD90, CD105), lack expression of hematopoetic markers, and remain cytogenetically stable at least until passage 6 [12].

### Cell stimulation

Passages 2 to 5 were used for experiments. For collagen and TGF-β1 secretion, mRNA and protein expression analyses as well as for signal transduction studies, 2x10^5^ cells were plated per 60-mm dish in complete α-MEM. The next day, medium was changed to experimental medium consisting of supplemented phenol red free DMEM (PAA) without PL or FCS. After 24 h, cells were changed to experimental medium containing either connective tissue growth factor (CTGF; Immunotools), basic fibroblast growth factor (b-FGF; Immunotools), platelet derived growth factor-BB (PDGF-BB; Sigma), or transforming growth factor-β1 (TGF- β1; R&D). Each growth factor was used at 10 ng/mL. Medium was changed after 3 days. After another 3 days, cell culture supernatants were collected, centrifuged at 10,000 g for 10 min and stored at -20°C after discarding the pellets.

### Phase contrast micrographs

Cultures were photographed after six days of growth factor (GF) stimulation using an inverted phasecontrast microscope (Zeiss) and a CCD camera (Canon). The original magnification was 200x. Representative images of six independent experiments are shown.

### Immunofluorescence

Cells grown on glass coverslips were fixed with ice cold methanol. Unspecific binding was blocked for 90 minutes at room temperature with 1% BSA in TBS. Anti-sm-α-actin antibody (clone 1A4, Sigma) was added 1:400 in blocking solution for 2 h at room temperature. After washing, an Alexa Fluor 488 labeled secondary antibody (1:500 in TBS) was used for detection (1 h at room temperature in the dark). Nuclei were stained with 4',6-diamidino-2-phenylindole (DAPI). Images were captured on a Zeiss Axioplan microscope.

### Protein extraction and western blotting

Cells in culture dishes were washed twice with PBS. 80 μl of ice cold lysis buffer (50 mM Tris, pH 7.5; 0.01% SDS; 0.5% Triton X-100; complete mini protease inhibitor cocktail (Roche); 10 mM NaF; 1 mM Na_3_VO_4_; 10 mM β-glycerophosphate; 2 mM sodium pyrophosphate) were added and cells were scraped into micro tubes. Lysates were passed five times through a 26-gauge needle, cleared by centrifugation at 10,000 g for 10 min at 4°C, and stored at –20°C until analysis. Total protein content was determined with the DC protein assay (Bio-rad, Munich, Germany).

Equal amounts of protein were separated by SDS-polyacrylamide gel electrophoresis under reducing conditions and transferred onto nitrocellulose membranes (Hybond-ECL, Amersham Biosciences, Freiburg, Germany) by electroblotting. Membranes were blocked with 5% non-fat dry milk in TBS containing 0.1% Tween 20 (TBST) for 1 h at room temperature. The following primary antibodies were diluted in blocking solution or 3% BSA/TBST and allowed to bind over night at 4°C: COL2A1 (clone M2139; Santa Cruz) 1:500, SM22α (polyclonal; Abcam) 1:10,000, sm-Calponin (clone hCP; Sigma) 1:1,000, myosin light chain kinase (clone K36; Sigma) 1:500, sm-α-actin (clone 1A4; Sigma) 1:20,000, pSMAD3-Ser423/425 (clone C25A9) 1:500, pAKT-Ser473 (clone D9E) 1:1,000, total AKT (polyclonal) 1:1,000, pERK1/2-Thr202/Tyr204 1:3,000 (clone D13.14.4E), total EKR1/2 1:1,000 (polyclonal; all from Cell Signaling Technology), TGFβ receptor I (clone V-22; Santa Cruz) 1:1,000, α-tubulin (DM1A; Sigma) 1:10,000, GAPDH (clone FL-335; Santa Cruz) 1:500. After incubation with the appropriate horseradish peroxydase-conjugated secondary antibodies (1:25,000 in TBST; Dianova), immunoreactive proteins were detected by chemiluminescence (Super Signal West regular, pico, or dura, Thermo Scientific). Band intensities were quantified using ImageJ 1.41i software.

### Ca^2+^ imaging

In six-well-plates, 75,000 cells per well were seeded onto glass coverslips in complete α-MEM and allowed to adhere overnight. Medium was switched to phenol red free DMEM without FCS or PL for 24 h. After treatment with GF in experimental medium for 6 days, cells were loaded with the Ca^2+^ indicator fluo-4-AM (Invitrogen, Karlsruhe, Germany) (10 μM) and pluronic acid (Merck, Darmstadt, Germany) (0.01%; w/v) for 30 min at room temperature in PSS (NaCl 134 mM, KCl 6 mM, CaCl_2_ 2 mM, MgCl_2_ 1 mM, HEPES 10 mM, glucose 10 mM, pH 7.4 with NaOH). Before taking records, the cells were washed with PSS and further incubated for 20 min to allow de-esterification of the dye.

Pretreatment with 1 μmol/L nimodipine (Sigma-Aldrich) was carried out for 5 minutes before adding KCl. Fluo-4 loaded cells were imaged using a BioRad MRC 1024 laser scanning confocal microscope attached to a Nikon Diaphot 300 inverted microscope. Excitation was performed at 488 nm and the emission wavelength was 500 nm. Images were collected at a rate of 1/second. Image processing was done using imageJ 1.41i (National Institutes of Health, USA, http://rsbweb.nih.gov/ij/). Background fluorescence was subtracted and changes in intracellular calcium were expressed as relative fluorescence changes, i.e. F/Fo (with Fo indicating the fluorescence before stimulation and F the time-dependent fluorescence signal after stimulation). Peak amplitudes of Ca^2+^ transients were calculated as (F_peak_-Fo)/Fo. Stock solutions of fluo-4 AM (2.5 mM) and of nimodipine (1 mM) were made using DMSO as solvent. High external potassium solutions were made by iso-osmotic substitution of NaCl with KCl in the PSS.

### Chemotaxis assay

The chemotaxis assay was performed using modified Boyden chambers (Neuro Probe 48-Well Micro Chemotaxis Chamber) with fibronectin-coated polyvinylpyrrolidone-free polycarbonate filter membranes with 8 μm pore size. The lower well of the Boyden chamber was loaded with chemoattractants (CTGF, b-FGF, PDGF-BB, TGF-β1) diluted in serum-free α-MEM to a final concentration of 5 ng/mL. Cells from either patients with SSc or healthy donors were cultured in α-MEM without PL or FCS overnight. Cells were trypsinized and the reaction was stopped by addition of soybean trypsin inhibitor. 25,000–30,000 cells were added in serum-free α-MEM to the upper well of the Boyden chamber, and migration was allowed for 25 h. After scraping off the cells that had not migrated, migrated cells were stained with DiffQuick (Dade Behring). Membranes were scanned, and cell migration was quantified by densitometry using ImageJ 1.41i software. Each independent experiment was performed in triplicate.

### Cell proliferation assay

5000 cells per well were seeded in 96-well plates in complete α-MEM and allowed to adhere overnight. The next day, medium was changed to α-MEM without FCS or PL. 24 h later, 10 ng/mL of CTGF, b-FGF, PDGF-BB, or TGF-β1 in α-MEM without FCS or PL were added together with BrdU (1:500). GF induced proliferation was measured after incubation for another 24 h as BrdU incorporation with the BrdU cell proliferation kit (Roche) following the manufacturer’s instructions. All measurements of each independent experiment were performed in five replicates.

### Quantification of collagen in cell culture supernatant (SirCol assay)

100 μl cell free supernatants were mixed with 1 ml 0.5 g/l Direct Red (Fluka) in picric acid (Roth) and incubated at room temperature for 30 min under constant agitation. After centrifugation (5 min, 12,000g), the pellet was washed twice with 1 ml 0.1 M HCl. After the last centrifugation step, the pellet was dried and reconstituted in 250 μl 0.5 M NaOH (Roth). Absorption was measured in an ELISA-plate-reader at 492 nm. The collagen amount was calculated using a standard curve derived from several dilutions of collagen I solution (1 mg/ml Biochrom) in experimental medium. Each independent experiment was measured in duplicate.

### Total RNA extraction, cDNA synthesis, and quantitative real-time PCR

Cells were scraped into 1 ml Isol-RNA Lysis Reagent (5Prime) on ice and transferred into 1.5 ml microtubes. After adding 0.2 ml chloroform (Merck), the samples were vigorously mixed for 30 sec, incubated at room temperature for 3 min, and centrifuged at 12,000g for 15 min at 4°C. 400 μl of the aqueous phase were transferred into a new microtube and thoroughly mixed with 0.5 ml isopropanol (Merck). Incubation at room temperature for 10 min was followed by another centrifugation step. After washing the pellet twice with 1 ml 70% ethanol, the pelleted RNA was dried and solved in 20 μl RNase free water.

The isolated RNA was quantified with a NanoDrop 1000 (Thermo Fisher Scientific) and adjusted to 0.1 μg/μl. Integrity was verified by electrophoretic separation of 200 ng RNA in 1% agarose gel. cDNA was synthesized from 500 ng RNA using a reverse transcription system (Applied Biosystems, Foster City, CA) with 2 μM random hexamers (Applied Biosystems) and 0.2 mM dNTP mix (Promega). Real-time PCR was performed in the 7500 Fast Real-Time PCR system (Applied Biosystems). The reaction was carried out in 20 μl reaction volumes including 2 μl of cDNA, primers (concentrations as given below; TIB MolBiol, Berlin, Germany) and 10 μl of Power SYBR Green PCR Master Mix (Applied Biosystems). The PCR program consisted of 2 min at 50°C, 95°C for 10 min followed of 40 cycles 95° for 15 sec and 1 min at 60°C. Specificity of the reaction was verified by melting curve analysis at the end of each series of assays.

All primers were designed according to the data given by the National Center for Biotechnology Information (NCBI) with assistance of the software Primer3 (http://primer3.sourceforge.net/releases.php). Quantification was performed via ΔΔCT-method in comparison to GAPDH as reference gene which was shown not to be regulated by the different treatments in extensive set up experiments. Primer sequences can be found in the online supplement.

### ELISA for TGF-β1

The concentration of TGF-β1 in cell culture supernatant was determined using the human TGF-β1 CytoSet (Invitrogen) following the manufacturer’s instructions. All samples were pre-treated with 1 M HCl for 15 min to convert TGF- β1 into its bioactive form. Each sample was analyzed in triplicate and quantified in Microsoft Office Excel by means of a corresponding standard curve with log-log curve fit.

### Statistics

All data are expressed as mean + SEM. 2-way ANOVA was performed to test for disease-growth factor-interaction and was followed by post-testing with Bonferroni's Test with correction for multiple comparisons (GraphPad Prism version 5.02 for Windows, GraphPad Software, San Diego California USA). Significance was considered at a value of p<0.05.

## Results

### Surface marker expression and multilineage differentiation potential of SSc-MSCs in comparison to H-MSCs

**Fig 1:**
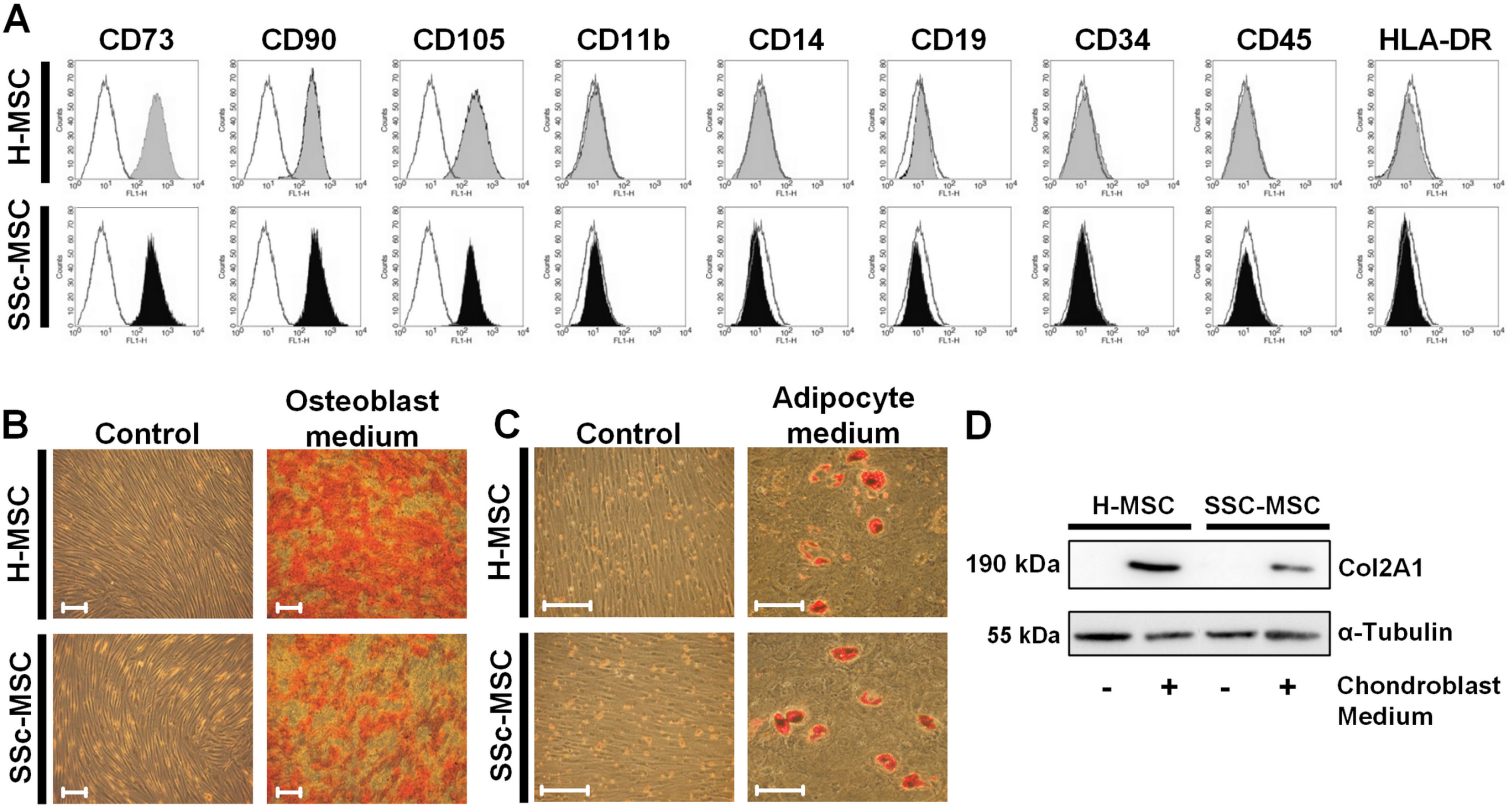
Characterization of mesenchymal stromal cells from healthy controls and patients with systemic sclerosis. **A,** Representative FACS analysis of an MSC defining surface marker panel confirming homogeneity of isolated cells. **B,** Osteoblastic differentiation of MSCs after incubation for 21 days with osteoblast-induction medium. (Alizarin red S staining, original magnification x100, scale bars = 50 μm) **C,** Adipocytic differentiation of MSCs after incubation for 21 days with adipocyte-induction medium. (Oil-Red-O staining, original magnification x200, scale bars = 50 μm) **D,** Chondroblastic differentiation of MSCs after incubation for 32 days with chondroblast induction medium. Western blot analysis for chondrocyte specific type II collagen.

MSCs from healthy controls and patients with SSc were isolated from bone marrow derived mononuclear cells by adherence to cell culture plastic. We obtained homogenous populations of H-MSCs and SSc-MSCs presenting the characteristic MSC surface marker profile with positivity for CD73, CD90 and CD105 whereas markers of hematopoietic lineages were negative (Fig 1A). We confirmed mesenchymal multilineage differentiation potential by induction of osteoblastic (Fig 1B), adipocytic (Fig 1C) and chondroblastic differentiation (Fig 1D) as required by the standard criteria for MSC research [13]. We did not detect any differences between MSCs from healthy donors and those from patients with SSc under basal conditions.

### MSC morphology and subcellular organization of the contractile cytoskeleton in response to SSc lesional growth factors

**Fig 2:**
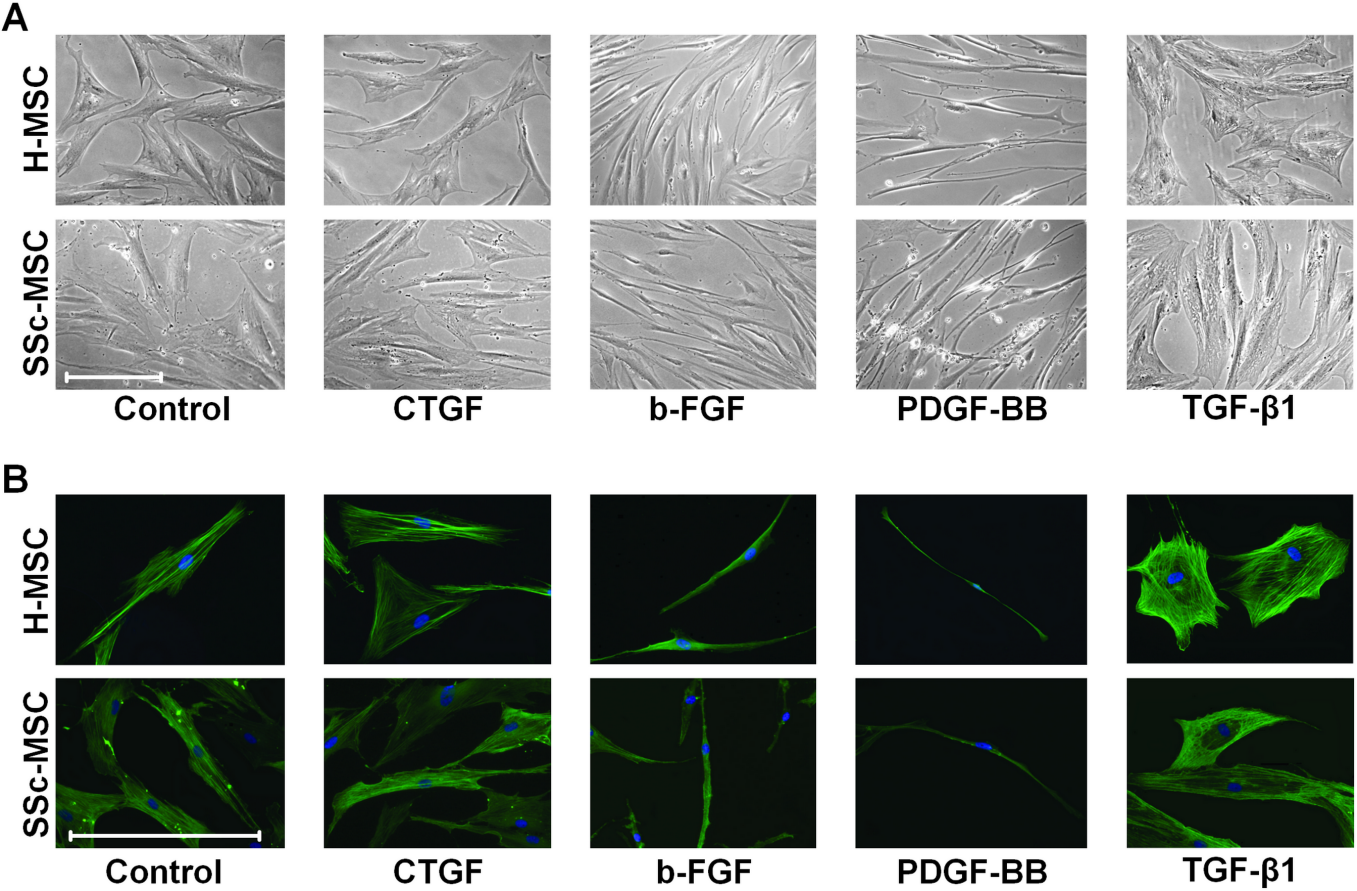
Phenotypic modulation upon long-term stimulation (6 days) with systemic sclerosis microenvironment defining growth factors. **A,** Representative phase contrast photomicrographs from a total of 6 independent experiments are shown. (original magnification x200, scale bar = 50 μm) **B,** Representative immunofluorescence images from a total of 6 independent experiments are shown. (green = smooth muscle-α-actin, blue = nuclear DNA stained with 4',6-diamidino-2-phenylindole (DAPI), original magnification x400, scale bar = 50 μm).

To test the hypothesis that disease specific alterations of the differentiation competence of a common mesenchymal precursor cell may contribute to development and progression of both, vasculopathy and fibrosis in SSc, we studied long-term treatment (6 days) of H-MSCs and SSc-MSCs with the SSc microenvironment-defining GFs CTGF, b-FGF, PDGF-BB, and TGF-β1. These GFs have been linked to vasculopathy as well as skin and organ fibrosis in SSc [1, 14]. Non-stimulated H-MSCs and SSc-MSCs had a similar microscopic phenotype characterized by an irregular shape and prominent sm-α-actin (SMA) positive stress fibers (Fig 2A and 2B) as we previously demonstrated for resting MSCs from healthy donors [11]. Size, shape, subcellular structure, and SMA expression in both, H-MSCs and SSc-MSCs remained similar to resting MSCs upon exposure to CTGF (Fig 2A and 2B). In response to b-FGF and PDGF-BB, H-MSCs and SSc-MSCs transformed into slim, elongated cells with considerably reduced cytoskeletal stress fibers. Only PDGF-BB treated cells developed multiple pointed extensions (Fig 2A and 2B). In contrast, TGF-β1 induced large cells with huge amounts of stress fibers resulting in a myofibroblast-like flattened appearance without processes (Fig 2A and 2B). H-MSCs and SSc-MSCs responded to the individual GFs with similar over-all microscopic changes.

### Deregulated phenotypic plasticity of SSc-MSC derived vascular smooth muscle cells and fibroblasts

**Fig 3:**
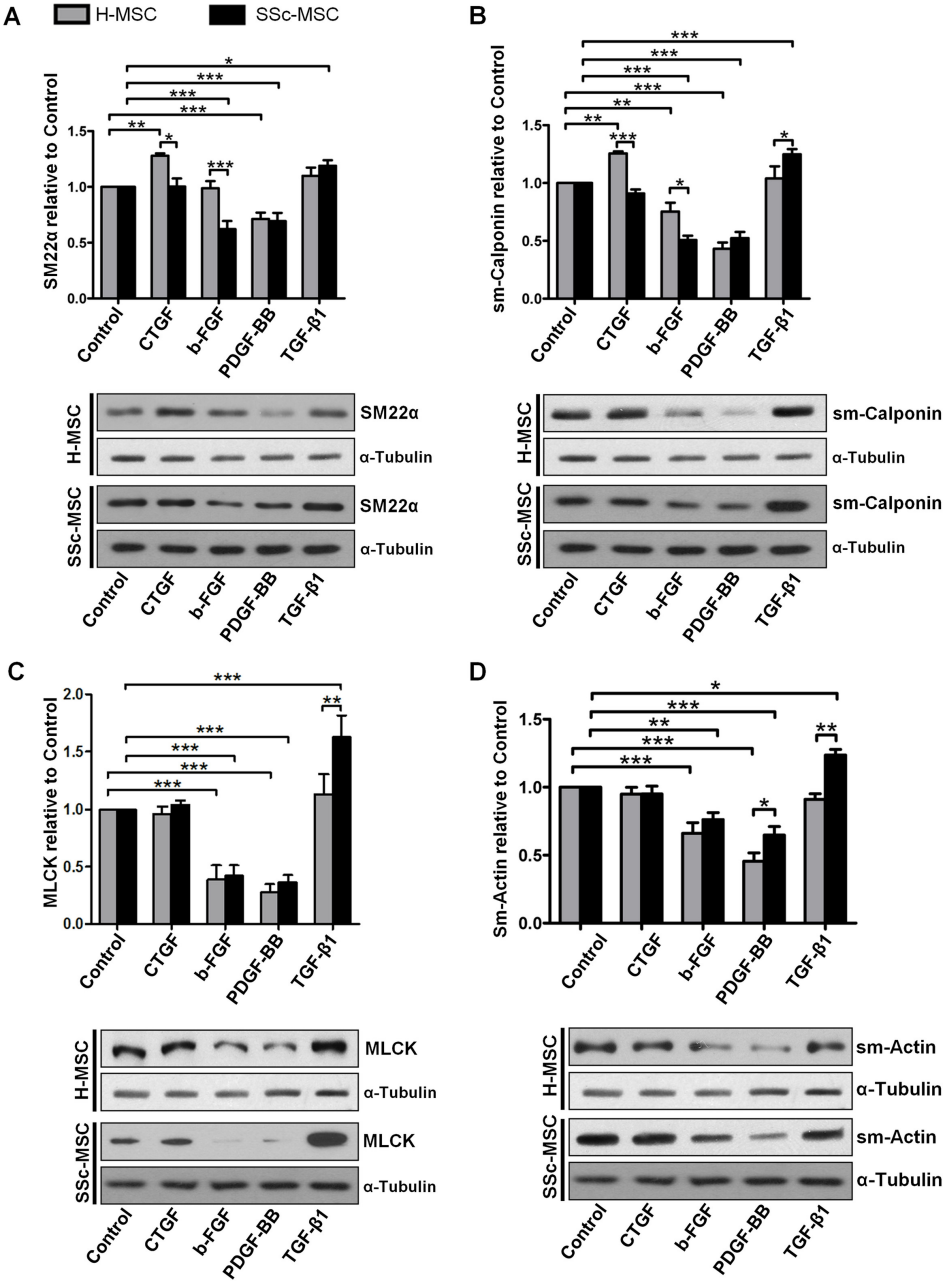
Expression of contractile proteins in response to treatment with systemic sclerosis microenvironment defining growth factors. **A,** SM22α **B,** smooth-muscle calponin (sm-Calponin) **C,** myosin-light-chain-kinase (MLCK) **D,** smooth muscle-α-actin (sm-Actin). Bars represent the mean+SEM of densitometrically determined band intensities after normalization to α-tubulin. Densitometric quantification and representative western blots of 6 independent experiments are shown. Cells hat been treated for 6 days. Control was set to 1. *P<0.05, **P<0.01, ***P<0.001.

Despite microscopic similarities, MSCs derived from SSc patients substantially differed from H-MSCs in disease relevant molecular and functional characteristics comprising VSMC differentiation. Compared to H-MSCs, SSc-MSCs displayed deregulation of the contractile VSMC program indicated by lack of up-regulation of terminal differentiation markers—SM22α and smooth muscle calponin (sm-Calponin)—in response to CTGF and by decrease in their expression upon b-FGF (Fig 3A and 3B). Expression of myosin light chain kinase (MLCK) was either unaffected upon CTGF or down-regulated by b-FGF in both cell types (Fig 3C). SMA down-regulation in response to b-FGF was similar in both groups (Fig 3D). In response to PDGF, both cell types down-regulated expression of all proteins characteristic for contractile VSMC phenotype (Fig 3A–3D) in a similar manner, as it is well established in the biology of primary VSMCs [15]. Only SMA levels were down-regulated to a lower extent in SSc-MSCs, as compared to H-MSCs (Fig 3D). In contrast to the other stimuli applied, TGF-β1 enhanced the expression of the analyzed contractile proteins especially of MLCK and SMA only in SSc-MSCs but not in H-MSCs (Fig 3A–3D), implicating a presumable induction of myofibroblast differentiation.

**Fig 4:**
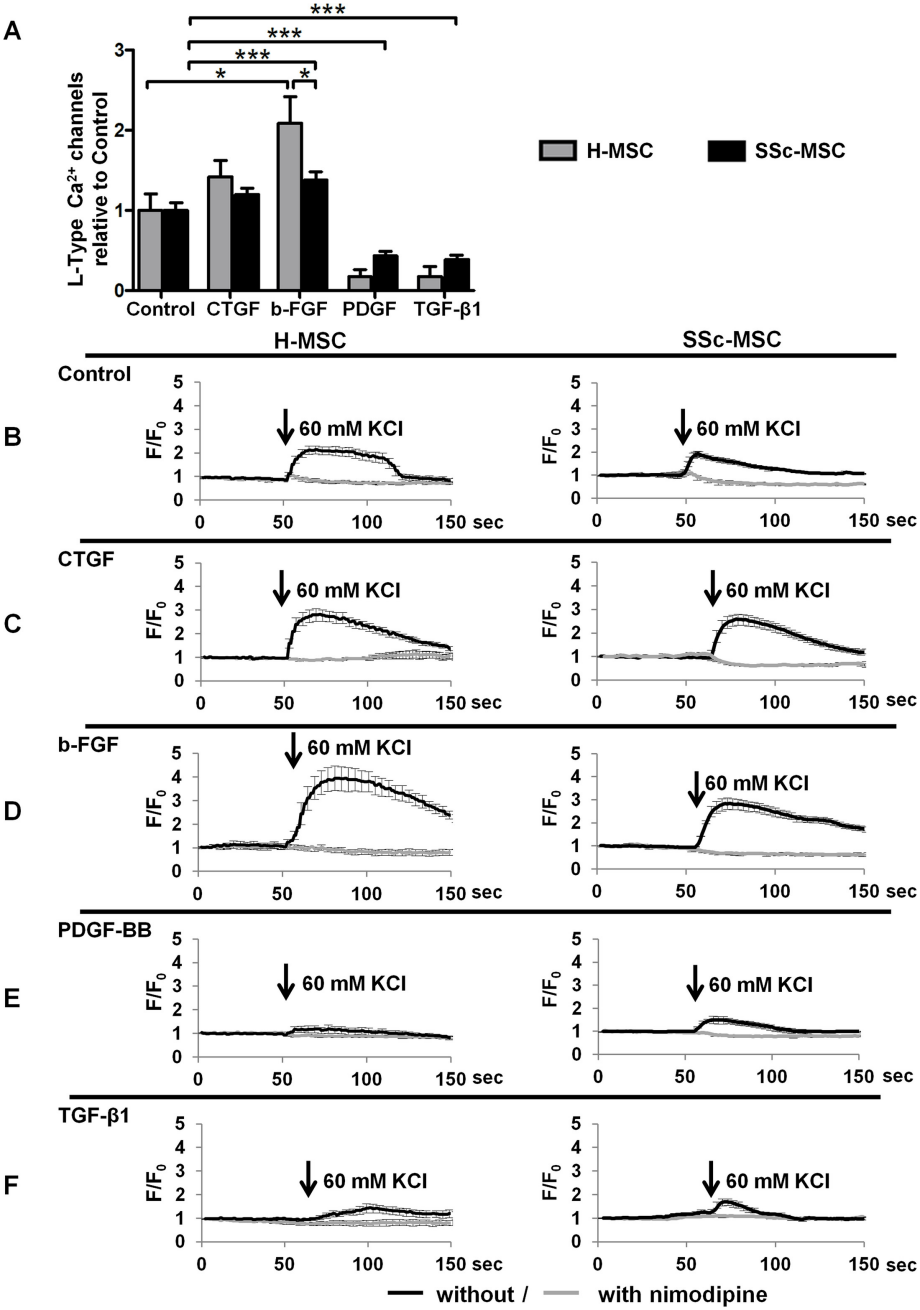
Measurement of functional L-type Cav1.2 Ca2+ channels in response to growth factors. Functional L-type Cav1.2 Ca^2+^ channels measured as nimodipine sensitive Ca^2+^-influx upon depolarisation with 60 mmol/L KCl in response to treatment with systemic sclerosis microenvironment defining growth factors for 6 days. **A,** Bars represent the mean+SEM of nimodipine sensitive Ca^2+^-influx from 3 independent experiments. Control was set to 1. *P<0.05, **P<0.01, ***P<0.001. **B-F,** Representative recordings of Ca^2+^ dependent intracellular fluorescence intensities in response to depolarisation with KCl. Mean±SEM F/F0 of 30–40 cells per growth factor are plotted against time. Black lines represent tracings without the specific L-type calcium channel blocker nimodipine, grey lines those obtained after preincubation with 1 mmol/L nimodipine.

VSMCs with contractile phenotype and myofibroblasts express a similar spectrum of contractile proteins, yet their functional response to extracellular depolarizing stimuli differs considerably. Hence, we measured functional L-type Cav1.2 Ca^2+^ channels, a hallmark of VSMC differentiation, using the specific L-type calcium channel blocker nimodipine. Under basal conditions, MSCs spontaneously differentiate towards contractile VSMC lineage and display functional L-type calcium channels (Fig 4A and 4B), as we reported previously [11]. CTGF maintained L-type calcium channels in MSCs of either origin (Fig 4A and 4C). In response to b-FGF, both cell types increased nimodipine sensitive Ca^2+^ influx indicating higher functional capacity of L-type calcium channels. This effect was significantly stronger in H-MSCs compared to SSc-MSCs (Fig 4A and 4D). In contrast, PDGF-BB and TGF-β1 almost abolished L-type calcium channel dependent intracellular increases in Ca^2+^ (Fig 4A, 4E and 4F) demonstrating loss of VSMC like differentiation.

**Fig 5:**
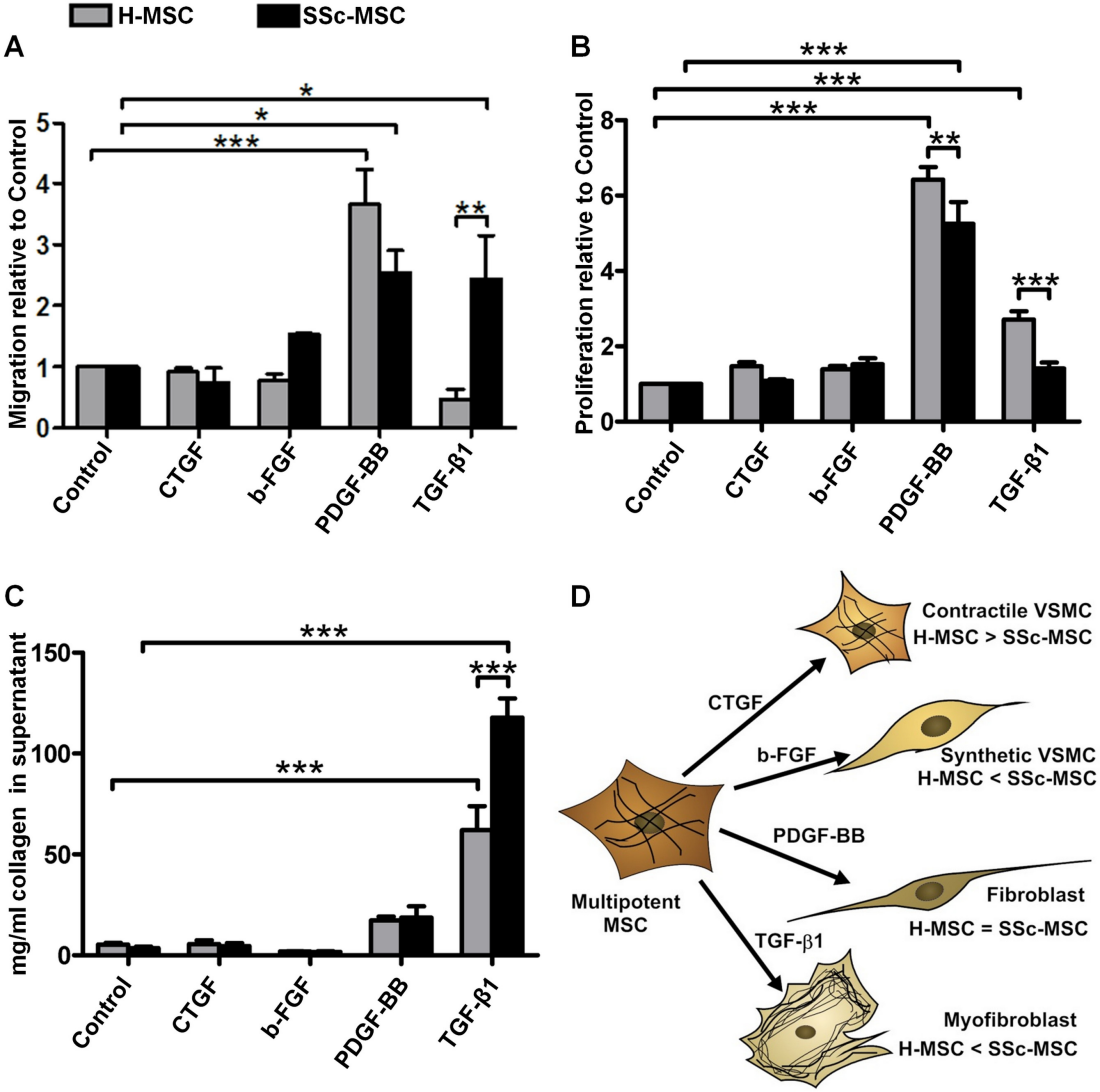
Cell functions of mesenchymal stromal cells upon treatment with systemic sclerosis microenvironment defining growth factors. **A,** Chemotactic response towards a 5 ng/ml gradient of each growth factor. Bars represent the mean+SEM of 3 independent experiments, 3 replicates each. Control was set to 1. **B,** Proliferation measured by BrdU incorporation after stimulation with 10 ng/ml of each growth factor for 24 h. Bars represent the mean+SEM of 3 independent experiments, 5 replicates each. Control was set to 1. **C,** Total collagen content in cell culture supernatants after 6 days of treatment with 10 ng/ml of each growth factor. Bars represent the mean+SEM of 6 independent experiments, 2 replicates each. *P<0.05, **P<0.01, ***P<0.001. **D,** Illustration of the induction of specific cell types of vascular smooth muscle cell (VSMC) and fibroblast lineages by systemic sclerosis microenvironment defining growth factors.

Contractile VSMCs contained in the tunica media of arteries respond to an injury induced release of GFs with a phenotypic switch towards the “synthetic” phenotype characterized by coordinated repression of the VSMC contractile gene program and concomitant increases in migration, proliferation, and production of extracellular matrix proteins [16]. In order to further discriminate between the differentiated contractile and the dedifferentiated synthetic state of VSMCs derived from MSCs, we assessed migration, proliferation and collagen production. Neither migration nor proliferation were induced by CTGF in H-MSCs or in SSc-MSCs (Fig 5A and 5B). B-FGF elicited a minor non-significant migratory response only in SSc-MSCs (Fig 5A). Secreted collagen was not altered by CTGF or b-FGF in both, H-MSCs and SSc-MSCs (Fig 5C). Taken together, CTGF promoted differentiation of VSMCs with a contractile phenotype, while b-FGF induced differentiation of VSMCs with a synthetic phenotype in H-MSCs. Both differentiation responses were deregulated in SSc-MSCs (Fig 5D).

PDGF-BB induced migratory and pro-proliferative responses in both, H-MSCs and SSc-MSCs, yet less accentuated in SSc-MSCs (Fig 5A and 5B). SSc-MSCs migrated towards a TGF-β1 gradient without increase in proliferation, while H-MSCs remained sedentary but proliferated effectively (Fig 5A and 5B). The increase in collagen content upon stimulation with PDGF-BB was moderate and without statistical significance (Fig 5C). Both cell types and especially SSc-MSCs responded to TGF-β1 with abundant collagen production (Fig 5C). Different functional phenotypes of H-MSCs and SSc-MSCs implicate that PDGF-BB and TGF-β1 induced two different MSC derived fibroblast populations (Fig 5D).

### Disturbed collagen homeostasis in activated myofibroblasts derived from SSc-MSCs

**Fig 6:**
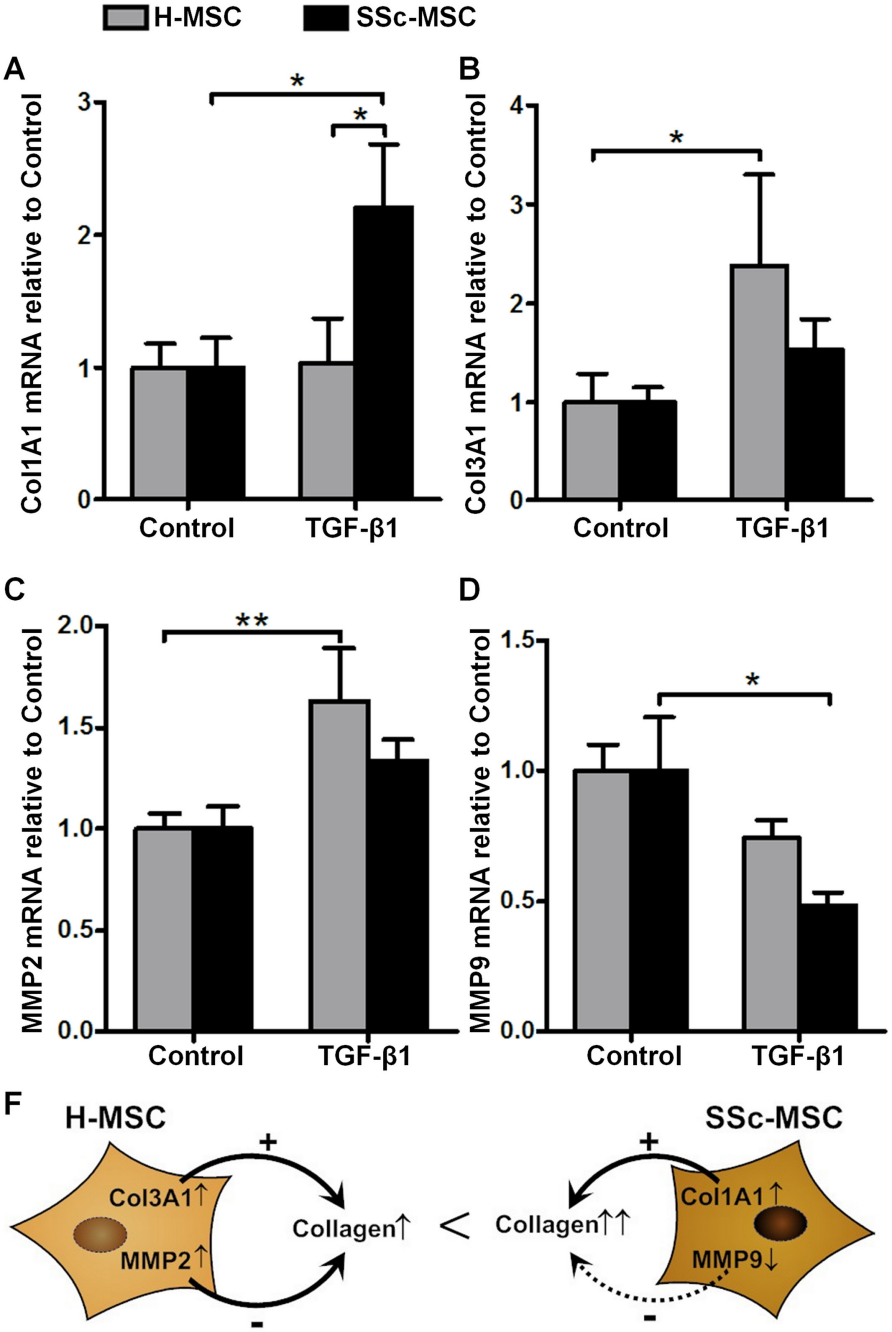
Measurement of collagen homeostasis as marker of fibrosis. Collagen homeostasis after 6 days of treatment with 10 ng/ml transforming growth factor-β1 in comparison to undifferentiated mesenchymal stromal cells. Quantitative real-time PCR analysis of mRNA transcripts for the collagen isoform defining chains Col1A1 (**A)** and Col3A1 **(B)** and the collagen degrading enzymes matrix metalloproteinase 2 **(C)** and matrix metalloproteinase 9 **(D)**. Bars represent the mean+SEM of 6 independent experiments quantified with the ΔΔCt method in duplicates. *P<0.05, **P<0.01. **E,** Scheme on how differentially regulated genes involved in collagen homeostasis may contribute to increased collagen secretion in systemic sclerosis.

SSc-MSCs responded to TGF-β1 with an increase in collagen type I α1 mRNA transcripts (Fig 6A), while H-MSCs increased collagen type III α1 levels (Fig 6B). Transcripts of the collagen degrading enzyme MMP2 were significantly elevated only in H-MSCs (Fig 6C), and MMP9 mRNA levels were significantly down-regulated only in SSc-MSCs (Fig 6D). We propose a model where TGF-β1 induced collagen synthesis together with insufficient up-regulation of MMP-2 and enhanced down-regulation of MMP-9 may result in the net effect of SSc related excessive collagen content in cultures of myofibroblasts derived from SSc-MSCs (Fig 6E).

### Hyperresponsive canonical and noncanonical TGF-β signaling in SSc-MSCs

**Fig 7:**
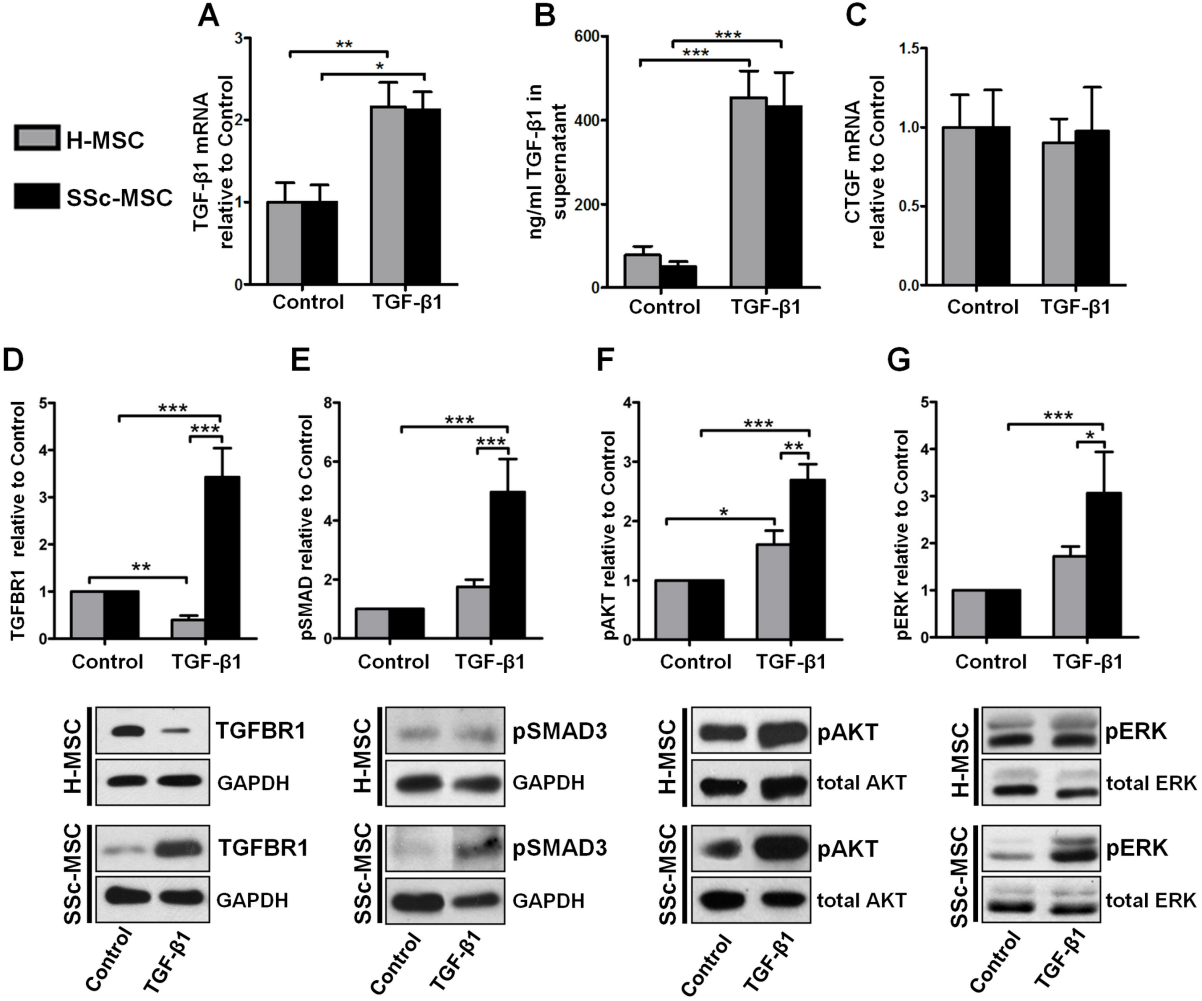
Analysis of the Transforming growth factor-β1 signaling network. **A,** Expression of TGF-β1 mRNA and **B,** secreted TGF-β1 protein for assessment of the autocrine TGF-β feedback loop. Bars represent the mean+SEM of 6 independent experiments, 3 replicates each. **C,** Expression of CTGF mRNA. Quantitative real-time PCR analysis of mRNA transcripts. Bars represent the mean+SEM of 6 independent experiments quantified with the ΔΔCt method in duplicates. **D,** Expression of TGF-β receptor 1 protein. **E-G,** Induction of canonic and non-canonic TGF-β signaling. **E,** Phosphorylation of SMAD3 at Ser423/425. **F,** Phosphorylation of AKT at Ser473. **G,** Phosphorylation of ERK1/2 at Thr202/Tyr204. Analysis of all experiments was performed after 6 days treatment with 10 ng/ml transformin growth factor -β1. Representative western blots of 6 independent experiments are shown. Bars represent the mean+SEM of densitometrically determined band intensities after normalization to GAPDH (TGFBR1, pSMAD3) or total kinase (pAKT, pERK). Control was set to 1. *P<0.05, **P<0.01, ***P<0.001.

Similar amounts of TGF-β mRNA (Fig 7A) and protein (Fig 7B) were produced by SSc-MSCs and H-MSCs implicating no differences between SSc-MSCs and H-MSCs in autocrine TGF-β loop regulation. Since CTGF may contribute to persistent TGF-β signaling in SSc [14, 17] we addressed this possibility in MSCs. We obtained no autocrine CTGF stimulation induced by TGF-β1 in SSc-MSCs or H-MSCs (Fig 7C). Similarly, neither expression of PDGF isoforms nor PDGF receptors were differentially regulated in H-MSCs and SSc-MSCs upon treatment with TGF-β1 (S1A–S1E Fig).

Focus on TGF-β related signal transduction pathways served to explain aberrant collagen homeostasis. A remarkably strong up-regulation of TGF-β receptor I (TGFBR1) protein in response to TGF-β1 was detected by western-blots only in SSc-MSCs derived myofibroblasts (Fig 7D). In clear contrast, TGFBR1 protein levels decreased in H-MSCs (Fig 7D). TGF-β1 had no influence on expression of TGF-β receptor II in H-MSCs and SSc-MSCs (S1F Fig). Furthermore, stronger induction of the canonical pro-fibrotic SMAD3 pathway demonstrated as an increase in SMAD3 phosphorylation by TGF-β1 was detected in SSc-MSCs as compared to H-MSCs (Fig 7E). AKT and ERK1/2, signaling components of noncanonical pathways involved in migration and proliferation, were also stronger activated in SSc-MSCs compared to H-MSCs (Fig 7F and 7G).

## Discussion

We report unique intrinsic alterations of MSCs from SSc patients in their response to key-mediators (CTGF, b-FGF, PDGF-BB, and TGF-β1) of SSc related vasculopathy and fibrosis. SSc microenvironmental GFs initiated distinct differentiation processes in MSCs ranging from VSMCs with contractile or synthetic phenotypes to two functionally different fibroblast populations. MSCs from SSc patients exhibited deregulated contractile VSMC phenoconversion and increased commitment towards myofibroblast differentiation. In comparison to H-MSCs, CTGF failed to promote a contractile VSMC phenotype in MSCs from SSc patients whereas the synthetic VSMC phenotype in response to b-FGF was facilitated. SSc-MSC derived myofibroblasts functionally resembled activated SSc lesional fibroblasts featuring increased collagen synthesis and enhanced migratory activity towards TGF-β1. Up-regulation and failure of ligand-dependent down-regulation of TGFBR1 accompanied by enhanced activation of canonical and non-canonical TGF-β signaling in SSc-MSCs may provide a responsible mechanism. Our data expand the recent findings of dysfunctional differentiation of SSc-MSCs towards the endothelial lineage [8] and broaden the concept of SSc-related impaired endogenous regenerative capacity of MSCs. This implies SSc as a prototypic disorder where disease initiation and progression are related to aberrant progenitor cell function.

Targeting cellular mediators of failed angiogenesis and uncontrolled fibrogenesis including progenitor cell-based therapies evolve as an important goal in the field of SSc. Exogenous cell therapies are increasingly facing biological challenges in achieving successful long-term regeneration [18]. Better understanding of repair barriers associated with disease-microenvironment related factors which may neutralize exogenously applied autologous progenitor cells receives increasing priority. We attempted to model the diseased tissue microenvironment by long-term stimulation of MSCs with prototypic GFs. We are aware that the number of patients included may represent a limitation of our study. However, all MSC preparations which were used displayed similar intrinsic biologic properties including constitutional hyperresponsiveness to TGF-β1. SSc-MSCs mounted uniform biologic responses in terms of all differentiation processes studied and consistent data were also reported in signaling experiments, independently whether the skin affection of the individual patient was limited or diffuse, whether the patient had been treated with glucocorticoids or not, or of patient age. Common disease denominators of all patients were digital ulcers and pulmonary fibrosis.

Despite some promising reports on autologous application of MSCs in SSc, our findings and those of others [19] raise concern about the safety of such strategies since maldifferentiation of MSCs can thwart their beneficial effects. If autologous MSCs are to be utilized for the treatment of SSc related complications, they may not only encounter a hostile microenvironment, but yet may also respond inappropriately to regenerative stimuli as our results suggest.

MSCs from SSc patients responded with impaired physiologic contractile VSMC differentiation to CTGF, and showed in parallel a synthetic phenotype in response to b-FGF. Our focus on deregulated plasticity of smooth muscle progenitors and thereby potentially disturbed arteriogenesis may provide an additional explanation related to the severity of vasculopathy in SSc as it expands the established concept of limited endothelial cell differentiation potential of SSc-MSCs [8]. Overall deregulation of a physiologic phenotypic switch of progenitor and resident cell types involved in vasculogenesis ranging from angiogenesis to arteriogenesis is plausible in SSc. Endothelial-to-mesenchymal phenotypic transition has also recently been implicated in the pathogenesis of SSc interstitial lung fibrosis [20]. Pericytes as integral cells of capillaries and microvessels of all organs have been implicated as a source of progenitor cells in vascular and tissue maintenance. Lately, it has become clear that pericytes are closely related to MSCs, as cultured pericytes and MSCs are indistinguishable regarding surface marker expression, biologic functions and in vitro differentiation capacity [21]. Moreover, pericytes carry MSC defining markers (CD44, CD90, CD73, CD105) in their in vivo niche [22]. These findings led to a model that considers pericytes and MSCs to represent different pools of the same cell—one attached to the abluminal side of endothelial cells, the other as “free” perivascular or circulating cells [23]. The local pericyte derived MSC pool can take part in tissue regeneration as has been suggested for myogenesis [22], fracture healing [24], and other conditions [25, 26]. On the other hand, pericytes can substantially contribute to organ damage. For example, fate tracing studies revealed that interstitial myofibroblasts in kidney fibrosis are derived from local pericytes [27]. In SSc, pericytes/MSCs have been linked to the development of fibrosis [28] and they may also be involved in tissue calcification [29]. Our results may additionally imply a role for SSc-pericytes/MSCs in obliteration of vessels by VSMC like derivatives. We are however aware that the notion of MSC/pericyte derived lesional cells in SSc in addition to those derived from residential VSMCs, fibroblasts, and circulating fibrocytes of hematopoietic origin cannot be sufficiently tested in patients in the near future due to exclusive applicability of fate-mapping studies to genetically modified mice [30].

Our final key finding was that TGF-β1 induced myofibroblast differentiation in SSc-MSCs compared to H-MSCs featured a clear functional dimorphism for almost all parameters studied including contractile cytoskeletal proteins, collagen homeostasis, and migration, which was similar to lesional fibroblasts. Likewise, fibroblasts isolated from affected tissue of SSc patients or from healthy controls differ substantially in biologic functions crucial for the development of the SSc phenotype. As an example, transcriptional regulation of genes involved in collagen homeostasis such as type I collagen, collagen degrading matrix metalloproteinase-9 (MMP-9), and the tissue inhibitor of metalloproteinases-1 (TIMP-1) are dysregulated in SSc lesional fibroblasts [31]. We found comparable disturbances in TGF-β1 induced SSc-MSCs resulting in accumulation of extracellular collagen.

Mechanisms responsible for tissue fibrosis in SSc are still incompletely understood and have been predominantly explained as an escape of SSc lesional fibroblasts to control mechanisms participating in physiologic wound healing [17]. Our study demonstrates that MSCs from SSc patients with skin and pulmonary fibrosis possess similar intrinsic biologic properties characterized by constitutional hyperresponsiveness to TGF-β1. SSc-MSCs retained high expression levels of TGFBR1 protein resulting in strongly induced canonical SMAD pathway and noncanonical ERK1/2 and AKT pathways persisting in SSc-MSCs even for 6 days after the initial stimulus. In contrast, H-MSCs showed ligand dependent TGFBR1 down-regulation and lack of hyperactivation of canonical and noncanonical signaling. Increased expression levels of TGF-β receptor II (TGFBR2) in MSCs from SSc patients under basal conditions and enhanced SMAD3 activation followed by increased collagen mRNA synthesis was recently independently reported upon short-term TGF-β1 stimulation (up to 24 h) [19]. Given these short-term stimulation data [19] and our long-term experiments, hyperresponsiveness of SSc-MSCs to TGF-β involves kinetic differences in TGFBR1 and TGFBR2. Short stimulation with low-concentration TGF-β1 (1 ng/ml for 6 and 24 h) induced TGF-β1 mRNA and protein only in SSc-MSCs but not H-MSCs [32], further emphasizing different kinetics in the autocrine TGF-β1 loop of SSc-MSCs and H-MSCs.

## Conclusions

In conclusion, our findings substantiate the concept of disease inherent abnormalities in differentiation capacity and biologic functions of MSCs from patients with SSc. Further refinements in the molecular understanding of disease-related functional diversity in MSC differentiation together with targeted approaches modulating disease-related local tissue microenvironment will be necessary to improve regenerative approaches in severe diseases such as SSc.

## Supporting Information

S1 Fig Analysis of PDGF-A-, C- and D-mRNA, PDGF-receptors and TGF-receptor expression. **A,** Expression of PDGF-A mRNA **B,** expression of PDGF-C mRNA**, C** expression of PDGF-D mRNA**, D,** expression of PDGF-Receptor A mRNA**, E,** expression of PDGF-receptor B mRNA, **F,** Expression of TGF-β-Receptor 2 mRNA. Bars represent the mean+SEM of 6 independent experiments quantified with the ΔΔCt method in duplicates. *P<0.05, **P<0.01, ***P<0.001.
(TIF)

## References

[1] Gabrielli A, Avvedimento EV, Krieg T. Scleroderma. The New England journal of medicine. 2009;360(19):1989–2003. 10.1056/NEJMra0806188 19420368

[2] Guiducci S, Giacomelli R, Cerinic MM. Vascular complications of scleroderma. Autoimmunity reviews. 2007;6(8):520–523. 17854742 10.1016/j.autrev.2006.12.006

[3] Pattanaik D, Brown M, Postlethwaite AE. Vascular involvement in systemic sclerosis (scleroderma). Journal of inflammation research. 2011;4:105–125. 10.2147/JIR.S18145 22096374 PMC3218751

[4] Trojanowska M. Cellular and molecular aspects of vascular dysfunction in systemic sclerosis. Nature reviews Rheumatology. 2010;6(8):453–460. 10.1038/nrrheum.2010.102 20585340 PMC3824624

[5] Del Papa N, Quirici N, Soligo D, Scavullo C, Cortiana M, Borsotti C, et al Bone marrow endothelial progenitors are defective in systemic sclerosis. Arthritis and rheumatism. 2006;54(8):2605–2615. 16868984 10.1002/art.22035

[6] Kirk TZ, Mark ME, Chua CC, Chua BH, Mayes MD. Myofibroblasts from scleroderma skin synthesize elevated levels of collagen and tissue inhibitor of metalloproteinase (TIMP-1) with two forms of TIMP-1. The Journal of biological chemistry. 1995;270(7):3423–3428. 7852429 10.1074/jbc.270.7.3423

[7] Larghero J, Farge D, Braccini A, Lecourt S, Scherberich A, Fois E, et al Phenotypical and functional characteristics of in vitro expanded bone marrow mesenchymal stem cells from patients with systemic sclerosis. Annals of the rheumatic diseases. 2008;67(4):443–449. 17526552 10.1136/ard.2007.071233

[8] Cipriani P, Guiducci S, Miniati I, Cinelli M, Urbani S, Marrelli A, et al Impairment of endothelial cell differentiation from bone marrow-derived mesenchymal stem cells: new insight into the pathogenesis of systemic sclerosis. Arthritis and rheumatism. 2007;56(6):1994–2004. 17530639 10.1002/art.22698

[9] Eyden B. The myofibroblast: phenotypic characterization as a prerequisite to understanding its functions in translational medicine. Journal of cellular and molecular medicine. 2008;12(1):22–37. 10.1111/j.1582-4934.2007.00213.x 18182061 PMC3823470

[10] Hegner B, Weber M, Dragun D, Schulze-Lohoff E. Differential regulation of smooth muscle markers in human bone marrow-derived mesenchymal stem cells. Journal of hypertension. 2005;23(6):1191–1202. 15894895 10.1097/01.hjh.0000170382.31085.5d

[11] Hegner B, Lange M, Kusch A, Essin K, Sezer O, Schulze-Lohoff E, et al mTOR regulates vascular smooth muscle cell differentiation from human bone marrow-derived mesenchymal progenitors. Arteriosclerosis, thrombosis, and vascular biology. 2009;29(2):232–238. 10.1161/ATVBAHA.108.179457 19074484

[12] Lange C, Cakiroglu F, Spiess AN, Cappallo-Obermann H, Dierlamm J, Zander AR. Accelerated and safe expansion of human mesenchymal stromal cells in animal serum-free medium for transplantation and regenerative medicine. J Cell Physiol. 2007;213(1):18–26. 17458897 10.1002/jcp.21081

[13] Dominici M, Le Blanc K, Mueller I, Slaper-Cortenbach I, Marini F, Krause D, et al Minimal criteria for defining multipotent mesenchymal stromal cells. The International Society for Cellular Therapy position statement. Cytotherapy. 2006;8(4):315–317. 16923606 10.1080/14653240600855905

[14] Varga J, Abraham D. Systemic sclerosis: a prototypic multisystem fibrotic disorder. The Journal of clinical investigation. 2007;117(3):557–567. 17332883 10.1172/JCI31139 PMC1804347

[15] Raines EW. PDGF and cardiovascular disease. Cytokine & growth factor reviews. 2004;15(4):237–254. 15207815 10.1016/j.cytogfr.2004.03.004

[16] Owens GK, Kumar MS, Wamhoff BR. Molecular regulation of vascular smooth muscle cell differentiation in development and disease. Physiological reviews. 2004;84(3):767–801. 15269336 10.1152/physrev.00041.2003

[17] Jinnin M. Mechanisms of skin fibrosis in systemic sclerosis. J Dermatol. 2010;37(1):11–25. 10.1111/j.1346-8138.2009.00738.x 20175837

[18] Bianco P, Cao X, Frenette PS, Mao JJ, Robey PG, Simmons PJ, et al The meaning, the sense and the significance: translating the science of mesenchymal stem cells into medicine. Nature medicine. 2013;19(1):35–42. 10.1038/nm.3028 23296015 PMC3998103

[19] Vanneaux V, Farge-Bancel D, Lecourt S, Baraut J, Cras A, Jean-Louis F, et al Expression of transforming growth factor beta receptor II in mesenchymal stem cells from systemic sclerosis patients. BMJ open. 2013;3(1). 10.1136/bmjopen-2012-001890 PMC3549232 23299111

[20] Mendoza FA, Piera-Velazquez S, Farber JL, Feghali-Bostwick C, Jimenez SA. Endothelial Cells Expressing Endothelial and Mesenchymal Cell Gene Products in Lung Tissue From Patients With Systemic Sclerosis-Associated Interstitial Lung Disease. Arthritis & rheumatology. 2016;68(1):210–217. 26360820 10.1002/art.39421 PMC4690777

[21] Crisan M, Corselli M, Chen CW, Peault B. Multilineage stem cells in the adult: a perivascular legacy? Organogenesis. 2011;7(2):101–104. 21593599 10.4161/org.7.2.16150 PMC3142446

[22] Crisan M, Yap S, Casteilla L, Chen CW, Corselli M, Park TS, et al A perivascular origin for mesenchymal stem cells in multiple human organs. Cell stem cell. 2008;3(3):301–313. 10.1016/j.stem.2008.07.003 18786417

[23] Bautch VL. Stem cells and the vasculature. Nat Med. 2011;17(11):1437–1443. 10.1038/nm.2539 22064433

[24] Caplan AI, Correa D. PDGF in bone formation and regeneration: new insights into a novel mechanism involving MSCs. Journal of orthopaedic research: official publication of the Orthopaedic Research Society. 2011;29(12):1795–1803. 21618276 10.1002/jor.21462

[25] Davidoff MS, Middendorff R, Enikolopov G, Riethmacher D, Holstein AF, Muller D. Progenitor cells of the testosterone-producing Leydig cells revealed. The Journal of cell biology. 2004;167(5):935–944. 15569711 10.1083/jcb.200409107 PMC2172461

[26] Olson LE, Soriano P. PDGFRbeta signaling regulates mural cell plasticity and inhibits fat development. Developmental cell. 2011;20(6):815–826. 10.1016/j.devcel.2011.04.019 21664579 PMC3121186

[27] Humphreys BD, Lin SL, Kobayashi A, Hudson TE, Nowlin BT, Bonventre JV, et al Fate tracing reveals the pericyte and not epithelial origin of myofibroblasts in kidney fibrosis. The American journal of pathology. 2010;176(1):85–97. 10.2353/ajpath.2010.090517 20008127 PMC2797872

[28] Rajkumar VS, Howell K, Csiszar K, Denton CP, Black CM, Abraham DJ. Shared expression of phenotypic markers in systemic sclerosis indicates a convergence of pericytes and fibroblasts to a myofibroblast lineage in fibrosis. Arthritis research & therapy. 2005;7(5):R1113–1123. 16207328 10.1186/ar1790 PMC1257439

[29] Collett GD, Canfield AE. Angiogenesis and pericytes in the initiation of ectopic calcification. Circulation research. 2005;96(9):930–938. 15890980 10.1161/01.RES.0000163634.51301.0d

[30] Zeisberg EM, Kalluri R. Origins of cardiac fibroblasts. Circulation research. 107(11):1304–1312. 10.1161/CIRCRESAHA.110.231910 21106947 PMC3098499

[31] Jimenez SA, Gaidarova S, Saitta B, Sandorfi N, Herrich DJ, Rosenbloom JC, et al Role of protein kinase C-delta in the regulation of collagen gene expression in scleroderma fibroblasts. The Journal of clinical investigation. 2001;108(9):1395–1403. 11696585 10.1172/JCI12347 PMC209435

[32] Guiducci S, Manetti M, Romano E, Mazzanti B, Ceccarelli C, Dal Pozzo S, et al Bone marrow-derived mesenchymal stem cells from early diffuse systemic sclerosis exhibit a paracrine machinery and stimulate angiogenesis in vitro. Annals of the rheumatic diseases. 2011;70(11):2011–2021. 10.1136/ard.2011.150607 21821866

