# Supplementary figures and images for: Intrinsic Deregulation of Vascular Smooth Muscle and Myofibroblast Differentiation in Mesenchymal Stromal Cells from Patients with Systemic Sclerosis
Source: PLoS One. 2016 Apr 7;11(4):e0153101. doi: 10.1371/journal.pone.0153101 (PMC4824407; doi:10.1371/journal.pone.0153101)

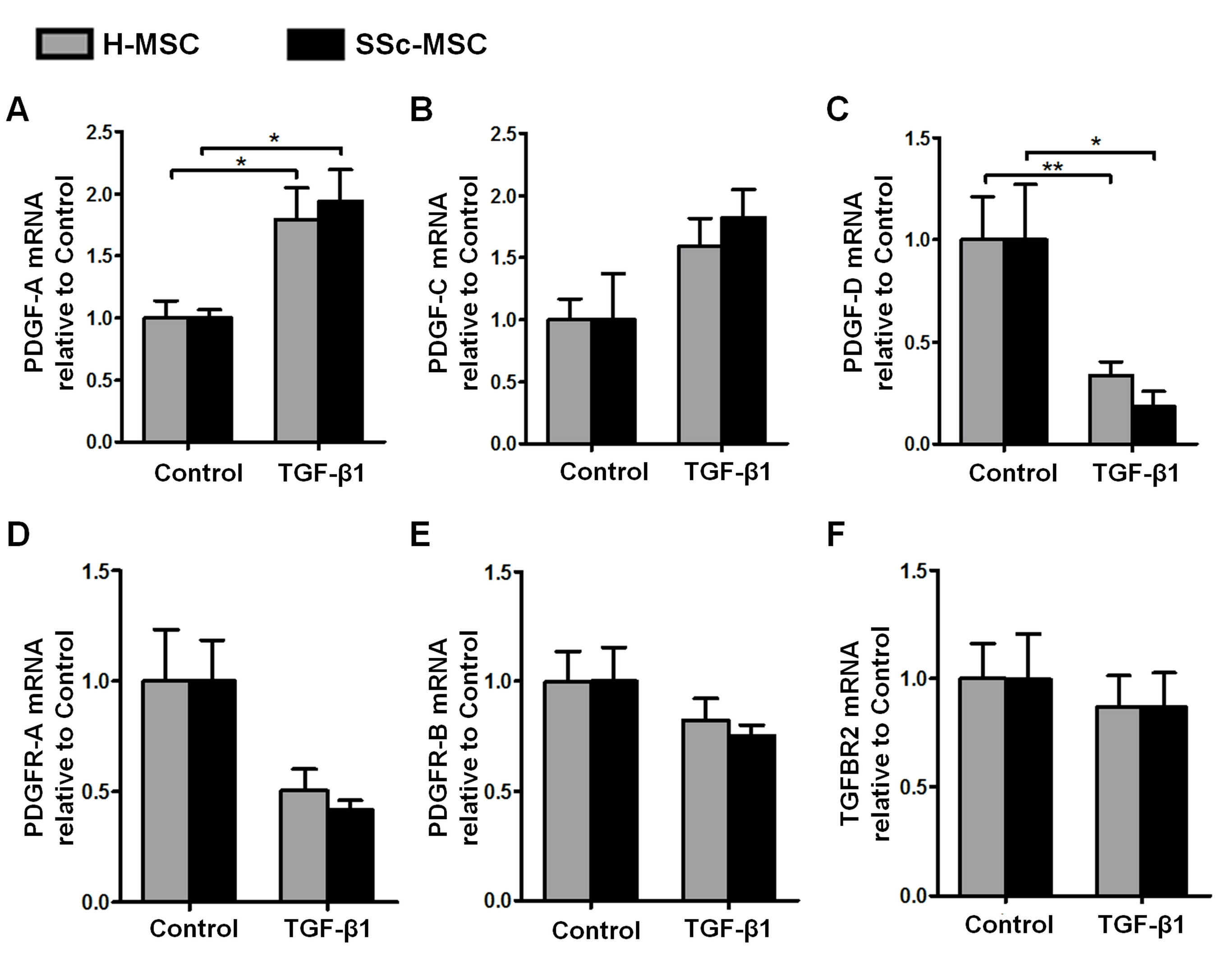

Supplement: S1 Fig — A, Expression of PDGF-A mRNA B, expression of PDGF-C mRNA, C expression of PDGF-D mRNA, D, expression of PDGF-Receptor A mRNA, E, expression of PDGF-receptor B mRNA, F, Expression of TGF-β-Receptor 2 mRNA. Bars represent the mean+SEM of 6 independent experiments quantified with the ΔΔCt method in duplicates. *P<0.05, **P<0.01, ***P<0.001. (TIF) [file pone.0153101.s001.tif]
